# Coinfection with influenza virus and non-typeable *Haemophilus influenzae* aggregates inflammatory lung injury and alters gut microbiota in COPD mice

**DOI:** 10.3389/fmicb.2023.1137369

**Published:** 2023-03-30

**Authors:** Xiao Wu, Run-Feng Li, Zheng-Shi Lin, Chuang Xiao, Bin Liu, Kai-Lin Mai, Hong-Xia Zhou, De-You Zeng, Sha Cheng, Yun-Ceng Weng, Jin Zhao, Rui-Feng Chen, Hai-Ming Jiang, Li-Ping Chen, Ling-Zhu Deng, Pei-Fang Xie, Wei-Min Yang, Xue-Shan Xia, Zi-Feng Yang

**Affiliations:** ^1^State Key Laboratory of Respiratory Disease, National Clinical Research Center for Respiratory Disease, Guangzhou Institute of Respiratory Health, The First Affiliated Hospital of Guangzhou Medical University, Guangzhou, China; ^2^Guangzhou Laboratory, Guangzhou, China; ^3^School of Pharmaceutical Science and Yunnan Key Laboratory of Pharmacology for Natural Products, Kunming Medical University, Kunming, China; ^4^Dongguan People’s Hospital, Dongguan, China; ^5^The Affiliated Anning First Hospital and Faculty of Life Science and Technology, Kunming University of Science and Technology, Kunming, China; ^6^Guangzhou Key Laboratory for Clinical Rapid Diagnosis and Early Warning of Infectious Diseases, Guangzhou, China; ^7^State Key Laboratory of Quality Research in Chinese Medicine, Macau University of Science and Technology, Taipa, Macau SAR, China

**Keywords:** COPD, influenza, *influenzae*, mice, inflammation, microbiota

## Abstract

**Background:**

Acute exacerbation of chronic obstructive pulmonary disease (AECOPD) is associated with high mortality rates. Viral and bacterial coinfection is the primary cause of AECOPD. How coinfection with these microbes influences host inflammatory response and the gut microbiota composition is not entirely understood.

**Methods:**

We developed a mouse model of AECOPD by cigarette smoke exposure and sequential infection with influenza H1N1 virus and non-typeable *Haemophilus influenzae* (NTHi). Viral and bacterial titer was determined using MDCK cells and chocolate agar plates, respectively. The levels of cytokines, adhesion molecules, and inflammatory cells in the lungs were measured using Bio-Plex and flow cytometry assays. Gut microbiota was analyzed using 16S rRNA gene sequencing. Correlations between cytokines and gut microbiota were determined using Spearman’s rank correlation coefficient test.

**Results:**

Coinfection with H1N1 and NTHi resulted in more severe lung injury, higher mortality, declined lung function in COPD mice. H1N1 enhanced NTHi growth in the lungs, but NTHi had no effect on H1N1. In addition, coinfection increased the levels of cytokines and adhesion molecules, as well as immune cells including total and M1 macrophages, neutrophils, monocytes, NK cells, and CD4 + T cells. In contrast, alveolar macrophages were depleted. Furthermore, coinfection caused a decline in the diversity of gut bacteria. *Muribaculaceae*, *Lactobacillus*, *Akkermansia*, *Lachnospiraceae*, and *Rikenella* were further found to be negatively correlated with cytokine levels, whereas *Bacteroides* was positively correlated.

**Conclusion:**

Coinfection with H1N1 and NTHi causes a deterioration in COPD mice due to increased lung inflammation, which is correlated with dysbiosis of the gut microbiota.

## 1. Introduction

Acute exacerbation is a catastrophic event during the course of chronic obstructive pulmonary disease (COPD) and contributes to COPD-related mortality (Hillas et al., 2016). In China, COPD patients experience between 0.5 and 3.5 acute exacerbations annually, which accounts for substantial economic burden. Infection is the most common cause of AECOPD, accounting for 70–85% of all cases (Leung et al., 2017). Approximately 50% of these patients have bacterial infections, such as *Haemophilus influenzae* (NTHi), *Streptococcus pneumoniae*, and *Staphylococcus aureus*, while 30% have respiratory virus infections, such as rhinovirus, parainfluenza virus, and influenza virus (Leung et al., 2017). Viruses are more likely to be detected in AECOPD patients with influenza-like symptoms (Biancardi et al., 2016). Bacteria could colonize in lower airways of COPD patients, which may stimulate a secondary infection after viral infection. Coinfection with virus and bacteria is also common in COPD, accounting for 6.5–27% of infection induced-AECOPD (Papi et al., 2006; Hewitt et al., 2016). Patients with AECOPD who had a coinfection had significantly worse lung function and required longer hospitalizations (Papi et al., 2006).

Clinical and animal studies have revealed a synergistic relationship between influenza virus infection and bacterial infection (Jones et al., 1983; Lee et al., 2010; Bosch et al., 2013). Influenza infection can increase susceptibility and severity to secondary bacterial infection (Lee et al., 2010). However, it is still unclear how coinfection influences host response in AECOPD. In addition, there is growing evidence that the gastrointestinal and respiratory tracts are inherently related, and that the interaction between the gut microbiota and host immunity influences disease progression (Budden et al., 2017; Qu et al., 2022). Patients with COPD or viral infection have a disturbed gut microbiota, which worsens pulmonary inflammation and disease severity (Yildiz et al., 2018; Li et al., 2021). Despite good adherence to GOLD Class D recommended anti-microbial therapy, many COPD patients still experience exacerbation, and customized treatment for high frequency exacerbators based on dynamic changes of microbiota is still lacking (Brennan et al., 2022). This limitation highlights the need for a better understanding of the host and microbial response in COPD with influenza and bacterial coinfection. In this study, we developed a COPD mouse model using an oral and nasal cigarette smoking (CS) exposure system, and further induced exacerbation by sequentially infecting the mice with influenza H1N1 virus and NTHi. We also determined viral and bacterial growth, cytokine response, adhesion molecule levels, the presence of inflammatory cells, and gut microbiome.

## 2. Materials and methods

### 2.1. Preparation of virus and bacteria

The influenza A/Puerto Rico/8/34 (PR8, H1N1) virus was obtained from the American Type Culture Collection (ATCC) and propagated in the allantoic cavities of 10-day-old embryonated SPF chicken eggs. The virus was tittered in MDCK cells (ATCC) using TCID_50_ (50% tissue culture infectious dose) assay calculated by Reed and Muench method (Reed and Muench, 1938).

The NTHi ATCC49766, a reference strain, was kindly provided by Professor Zhuo from our laboratory. Due to the infectivity and virulence in mice (Kimura et al., 2020), we selected this strain for studies of dual infection-induced AECOPD. NTHi stocks were recovered in chocolate agar plates containing 0.33 mg/L vancomycin (Guangdong Huankai, China) and grown in brain heart infusion (BHI). 100 μL serial dilutions of NTHi in PBS were inoculated in chocolate agar plates for 24 h in a 37°C, 5% CO_2_ incubator, after which the colony-forming units (CFU) were determined. All infections experiments were conducted in a biosafety level 2 Plus (BSL-2 +) laboratory following the protocols approved by Guangzhou Medical University.

### 2.2. Mouse model of coinfection-induced AECOPD

Wild-type C57BL/6N male mice (6–8 weeks old), were purchased from Beijing Vital River Laboratory Animal Technology Co., Ltd. (Beijing, China). All the mice were housed in the specific pathogen-free facilities and all experimental protocols were approved by the Animal Care and Use Committee of Kunming Medical University. All mice were allowed to acclimatize for a week prior to cigarette smoke (CS) exposure.

**FIGURE 1:**
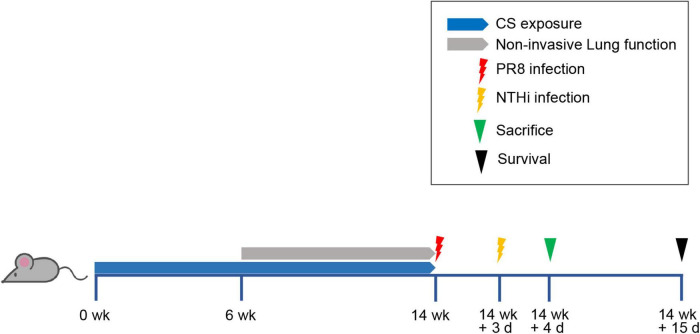
Experimental flow chart. Mice were exposed to one cigarette per day for 14 weeks and then intranasally inoculated with non-lethal dose of PR8 or PBS. At 3 days post-infection (dpi), mice were challenged with NTHi or PBS. After detecting invasive lung function at 24 h post-NTHi challenge, mice were sacrificed and samples were collected for further analysis, including histopathology, lung index, replication of pathogens, inflammatory mediators, immune cells, and gut microbiota. Survival rate was calculated at day 15 after PR8 infection.

The mice were randomly divided into 5 groups: CS exposure (Group 1), air exposure (Group 2), CS exposure with PR8 infection (Group 3), CS exposure with NTHi infection (Group 4), and CS exposure with PR8 and NTHi coinfection (Group 5). The experimental flow chart was shown in Figure 1. Briefly, group 1, 3, 4, and 5 were exposed to one cigarette (Hongtashan, China) per day for 14 weeks in an oral and nasal exposure system (Beijing Huironghe Technology, China), as previously described (Xiao et al., 2021). Each cigarette yielded 10 mg tar, 11 mg carbon monoxide, and 1.0 mg nicotine. The parameters of exposure were as followed: concentration of oxygen, (20 ± 0.5) %; dilution flow, 10 L/min; air extraction flow, 13 L/min; air humidity, (60 ± 5)%. The cigarette suction parameters were as followed: suction time, 2 s; time interval, 2 s; suction frequency, 10 s; suction volume, 35 mL. At week 14 post-CS exposure, mice from group 3 and 5 were anesthetized with 2.5% isoflurane and intranasally infected with 0.5 LD_50_ of PR8. After 3 days, group 5 were sequentially challenged with 10^5^ CFU of NTHi. These infectious doses have been established in our previous study of PR8-NTHi coinfection (Wu et al., 2021). At 24 h after NTHi challenge, the invasive lung function was measured. The mice were then sacrificed and the lungs were collected. The whole lungs were weighted and the lung index (lung weight/body weight ratio × 100) was calculated. Left lungs were then rinsed with HBSS containing 2% FBS before flow cytometric analysis of immune cells. Right lungs were homogenized in 1 mL PBS, and 100 μL of homogenate was used for bacterial titer determination. The remaining homogenates were further centrifugated at 3,000 rpm, 4°C, for 10 min, and the resultant supernatant was subjected to measurements of virus titer and cytokine levels. Also, whole lungs were harvested for a hematoxylin and eosin (H and E) staining. In addition, colon luminal content was collected for gut microbiota analysis. In survival study, the body weight and survival of mice were recorded daily for 15 days after PR8 infection.

### 2.3. Pulmonary function measurement

Pulmonary function in conscious mice was determined once every 2 weeks from week 6 to 14 after initiation of CS exposure using whole body plethysmography (EMKA Technologies, Canada). The spirometric parameters included frequency (F), tidal volume (TV), expiratory volume (EV), and inspiratory time (Ti).

Invasive lung function of mice was measured by using FlexiVent system (SCIREQ, Canada) according to the manufacturer’s protocol. Briefly, after anesthetization with tribromoethanol (Avertin, 240 mg/kg), mice were tracheotomized, intubated, and placed in the chamber of the FlexiVent system. To maintain muscular relaxation, mice were given intraperitoneal injections of vecuronium bromide (6 mg/kg). Mechanical ventilation was subsequently initiated to measure lung function determined by the forced vital capacity (FVC), the forced expiratory volume in 0.1 s (FEV0.1), respiratory resistance (Rrs), tissue damping (G), and respiratory system compliance (Crs). Each measurement was performed in triplicate. The data were analyzed by the FlexiVent software (SCIREQ, Montréal, QC, Canada).

### 2.4. Histopathology

The lung histopathology was performed as previously described (Shen et al., 2014). Briefly, the whole lungs of mice were removed and fixed in 4% paraformaldehyde for 24 h. After dehydration and embeddedness, lungs were sectioned to 3 μm thickness and stained with H and E or Masson’s trichrome. The histopathological lesions were observed and recorded under a light microscope. Trichrome staining intensity of 4 randomly selected areas per mouse lung was analyzed using ImageJ software.

### 2.5. Bacterial and viral titer determination

*Haemophilus influenzae* were quantified by incubating 100 μL of 10-fold serial dilutions of lung homogenates in chocolate agar plates containing vancomycin, as described above. After centrifugation of the remaining lung homogenates, supernatants were used to determine TCID_50_ in MDCK cells.

### 2.6. Cytokine and adhesion molecule measurement

The supernatants of lung homogenates were also used for the measurement of inflammatory cytokines, chemokines, and adhesion molecules. The cytokines and chemokines were determined by using the Luminex ProcartaPlex kit (Invitrogen, Waltham, MA, USA) and the Mouse magnetic Luminex assays kit (R&D, Santa Clara, CA, USA) in a Bio-Plex 200 Multiplex Testing System (Bio-Rad, Hercules, CA, USA) following manufacturer’s instructions. Mouse carcinoembryonic adhesion molecule-1 (CEACAM-1) (MyBioSource, San Diego, CA, Canada), intracellular adhesion molecules-1 (ICAM-1) (R&D), and fibronectin (Fn) (Abnova, Walnut, CA, USA), were determined by ELISA assays.

### 2.7. Flow cytometric analysis

The collected left lung was digested by the enzyme mixture in the gentleMACS Dissociator (Miltenyi Biotec, Gaithersburg, MD, USA). The resultant digested fluid was allowed to run through a 70-micron cell strainer to obtain a single-cell suspension. The erythrocytes were further lysed in a red blood cell lysis buffer (Biolegend, San Diego, CA, USA). Cells were resuspended with 1 mL FACS buffer (PBS contained 2% FBS and 5 mM EDTA), and the Fc receptors were blocked by anti-mouse CD16/32 (Biolegend, San Diego, CA, USA) at 4°C for 10 min. Cells were then stained with special anti-mouse antibodies, including GhostDye510 (TONBO Biosciences, USA), FITC anti-mouse CD11b (Biolegend, San Diego, CA, USA, Clone M1/70), PerCP-Cy5.5 anti-mouse Ly-6C (Biolegend, San Diego, CA, USA, Clone HK1.4), PE anti-mouse F4/80 (Biolegend, San Diego, CA, USA, Clone BM8), PE-Cy7 anti-mouse CD86 (Biolegend, San Diego, CA, USA, Clone GL-1), APC anti-mouse Ly-6G (Biolegend, San Diego, CA, USA, Clone 1A8), APC-Cy7 anti-mouse CD45 (Biolegend, San Diego, CA, USA, Clone 30-F11), BV421 anti-mouse CD11c (Biolegend, San Diego, CA, USA, Clone N418), BV605 anti-mouse CD206 (Biolegend, San Diego, CA, USA, Clone C068C2), FITC anti-mouse CD4 (Biolegend, San Diego, CA, USA, Clone RM4-5), PerCP-Cy5.5 anti-mouse CD3 (Biolegend, San Diego, CA, USA, Clone 17A2), PE anti-mouse CD19 (Biolegend, San Diego, CA, USA, Clone 6D5), APC anti-mouse CD8 (Biolegend, San Diego, CA, USA, Clone 53-6.7), and BV421 anti-mouse NK1.1 (Biolegend, San Diego, CA, USA, Clone PK136). Flow cytometric analysis of stained cells were performed using a flow cytometer (PARTEC CyFlow Space, Germany). Acquired data were analyzed using FlowJo software.

### 2.8. Gut microbiota analysis

Colon luminal contents in mice were collected immediately after euthanasia and were rapidly flash-frozen in liquid nitrogen for 30 min before storing at −80°C. Total bacterial DNA was extracted by using a Magnetic soil and stool DNA kit (TIANGEN, China). The V3-V4 region of 16S rRNA gene was amplified using PCR assay with. The amplicon primer sequences were as follows: forward primer 5′-(GTGCCAGCMGCCGCGGTAA)-3′; reverse primer 5′-(GGACTACHVGGGTWTCTAAT)-3′. The libraries were constructed using NEB next ultra-library prep kit (Illumina, USA) and paired-end sequenced on Illumina Novaseq6000 platform (Illumina, USA) of Novogene Co., Ltd. (Beijing, China). The filtering of raw sequence and taxonomic classification were performed as described previously (Li et al., 2020). The diagrams of alpha and beta diversity and relative abundance of microbiota were drawn using the R packages ggplot2 and vegan. The significance of beta diversity based on Weighted UniFrac dissimilarity was tested by analysis of similarities (Anosim) within the R package vegan.

### 2.9. Statistical analysis

Statistical analysis was performed by using GraphPad Prism 8.0 and SPSS 16.0 software. The differences among groups were compared by using the unpaired two-tailed student’s *t*-test, welch’s *t*-test or non-parametric test. Correlations between cytokines and gut microbiota were assessed by using Spearman’s rank correlation. The value of *p* < 0.05 was considered statistically significant.

## 3. Results

### 3.1. Coinfection with PR8 and NTHi results in higher mortality in COPD mice

**FIGURE 2:**
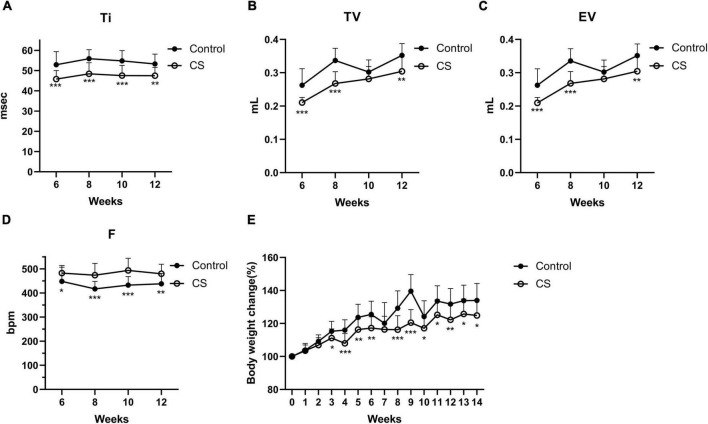
Lung function and body weight changes in conscious mice during CS exposure. Inspiratory time (Ti) **(A)**, tidal volume (TV) **(B)**, expiratory volume (EV) **(C)**, frequency (F) **(D)**, and body weight changes **(E)** in control (*n* = 21) and CS-exposed (*n* = 16) group. Data was expressed as means ± SD. *p < 0.05, **p < 0.01, and ***p < 0.001, compared with air exposure group.

**FIGURE 3:**
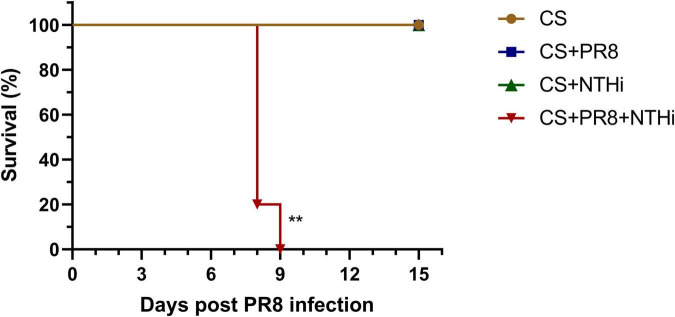
Lethal synergistic effect of PR8-NTHi coinfection in COPD mice. Three days following intranasal infection with 1 LD_50_ of PR8, COPD mice were challenged with 10^5^ CFU of NTHi. Survival (*n* = 5) was recorded for 15 days. **p < 0.01 compared with CS-exposed group.

To replicate COPD in humans, we first developed a mouse model by CS exposure for 14 weeks. Mice exposed to CS had impaired lung function, as indicated by an increase in F and a decrease in Ti, TV, and EV (Figures 2A–D). Besides, CS-exposed mice gained weight significantly slower than control mice (Figure 2E). These results suggested that COPD mice were successfully established. We further developed the AECOPD model by sequentially infecting COPD mice with PR8 and NTHi. Coinfected mice exhibited 100% lethality (*p* < 0.001), but PR8 or NTHi alone had no effect on mortality, indicating that the combination of two pathogens caused synergistic mortality (Figure 3).

### 3.2. Coinfection results in declined lung function in COPD mice

**FIGURE 4:**
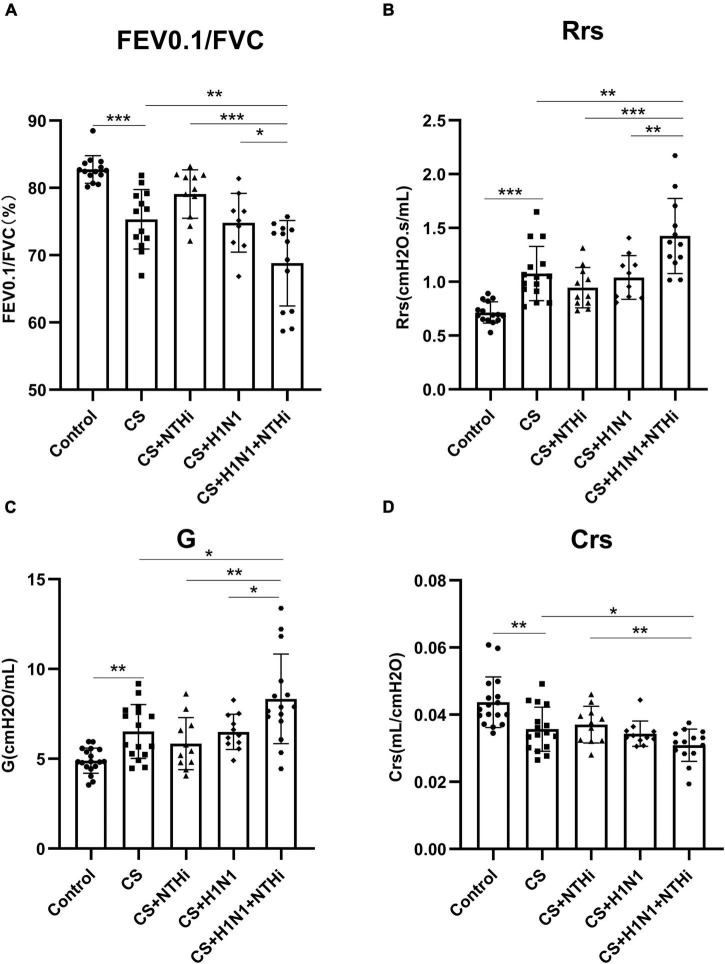
Coinfection with PR8 and NTHi impaired invasive lung function in AECOPD mice. At 24 h after NTHi challenge, invasive lung function was measured by forced expiratory volume in 0.1 s/forced vital capacity (FEV0.1/FVC) **(A)**, respiratory resistance (Rrs) **(B)**, tissue damping (G) **(C)**, and respiratory system compliance (Crs) **(D)**. Data are expressed as mean ± SD (*n* = 10–15). *p < 0.05, **p < 0.01, and ***p < 0.001.

As expected, CS exposure significantly impaired lung function, as shown by a decline in FEV0.1/FVC and Crs, and a increase in Rrs and G compared to control mice (Figures 4A–D), but PR8 or NTHi alone did not result in further impairment (Figures 4A–D). Interestingly, coinfection deteriorated the impairment of lung function in COPD mice (Figures 4A–D).

### 3.3. PR8 enhances NTHi growth in lungs of COPD mice

**FIGURE 5:**
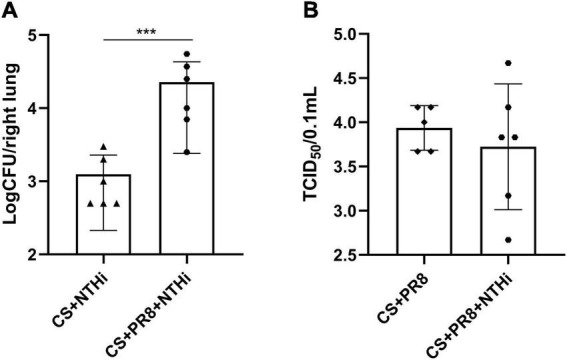
Replication of PR8 and NTHi in AECOPD mice. At 24 h post-NTHi infection, lung homogenates were added to chocolate agar plates containing vancomycin to determine bacterial CFU **(A)**, and the supernatants of lung homogenates were added to MDCK cells to determine viral TCID_50_ **(B)**. Data are expressed as mean ± SD (*n* = 6). ***p < 0.001.

The combination of PR8 and NTHi resulted in significant higher levels of NTHi load in mice lungs at 24 h post-NTHi challenge (Figure 5A), but the PR8 titer in coinfected mice did not differ from that in PR8-infected mice (Figure 5B).

### 3.4. Coinfection aggravates lung injury in COPD mice

**FIGURE 6:**
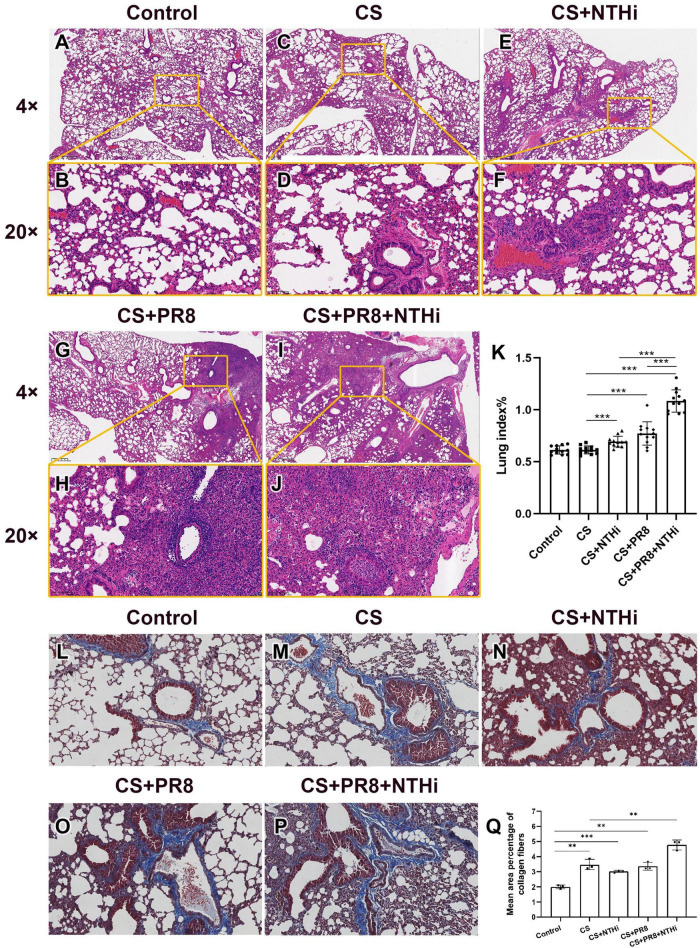
Coinfection aggravates lung injury in AECOPD mice. Pathological changes of control group **(A,B)**, CS-exposed group **(C,D)**, CS + NTHi group **(E,F)**, CS + PR8 group **(G,H)**, and CS + PR8 + NTHi group **(I,J)** were observed in H and E-stained sections. Original magnification, × 4 and × 20. Lung index was calculated as lung weight/body weight × 100 **(K)**. Collagen fibers of control **(L)**, CS-exposed **(M)**, CS + NTHi **(N)**, CS + PR8 **(O)**, and CS + PR8 + NTHi **(P)** groups were stained with Masson’s trichrome and quantified using ImageJ **(Q)**. Data are representative images (*n* = 3 in each group) or expressed as mean ± SD (*n* = 12 for lung index, *n* = 3 for percentage of collagen fibers). ^**^p < 0.01 and ^***^p < 0.001.

Mice exposed to CS had emphysema-like pathological changes, including bronchial wall thickening, alveolar wall thinning and destruction, and alveolar enlargement below the pleura, as compared with control group (Figures 6A–D). Mild inflammation was observed in COPD mice infected with NTHi, as shown by the recruitment of neutrophils into bronchial lumen (Figures 6E, F). In PR8-infected COPD mice, lymphocytic inflammation was present in peribronchial alveolar (Figures 6G, H). In addition, coinfected mice had more severe lung injury, as evidenced by infiltration of neutrophils into bronchiolar cavity, profuse hemorrhage and edema, and destruction of ciliated columnar epithelium (Figures 6I, J). In accordance with histopathological findings, a single pathogen resulted in a higher lung index than CS exposure alone, and coinfection resulted in the highest lung index (Figure 6K). In addition, mild collagen deposition was observed in smooth muscle layer of trachea and lung blood vessels of normal control mice (Figures 6L, Q). Compared to control group, mice exposed to CS exhibited bronchial wall thickening and significantly increased collagen deposition (Figures 6M, Q). Collagen expression was not significantly different between the CS, CS + NTHi, and CS + PR8 groups (Figures 6M, N, O, Q), but it was more extensive and significant in coinfected mice (Figures 6P, Q). These findings indicate that coinfection significantly increases the collagen deposition in lungs of COPD mice.

### 3.5. Coinfection results in increased inflammation in lungs of COPD mice

**FIGURE 7:**
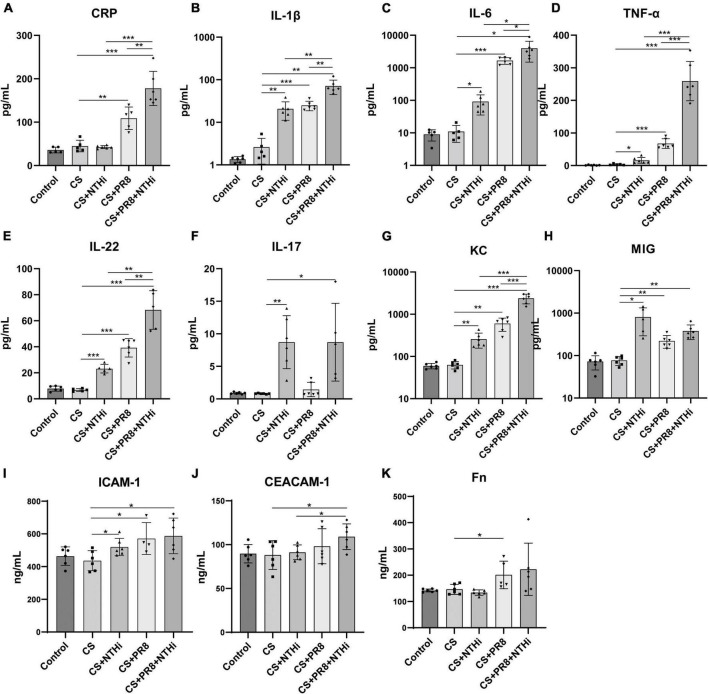
Cytokine and adhesion molecule expression in lung homogenates of AECOPD mice. The levels of CRP **(A)**, IL-1β **(B)**, IL-6 **(C)**, TNF-α **(D)**, IL-22 **(E)**, IL-17 **(F)**, KC **(G)**, MIG **(H)**, ICAM-1 **(I)**, CEACAM-1 **(J)**, and Fn **(K)** were measured by the Bio-Plex Mouse Cytokines assay or ELISA. Data are expressed as mean ± SD (*n* = 6 in each group). *p < 0.05, **p < 0.01, and ***p < 0.001.

Since pathological inflammation was pronounced in AECOPD mice, we further determined the expression levels of inflammatory cytokines and immune cells in the lungs. NTHi alone induced increased levels of cytokines, including IL-1β, IL-6, TNF-α, IL-22, IL-17, KC, and MIG, as compared with non-infected COPD mice (Figures 7B–H). Besides, PR8 infection alone also triggered significantly higher expression of inflammatory cytokines and chemokines, including CRP, IL-1β, IL-6, TNF-α IL-22, IL-17, KC, and MIG (Figures 7A–H). Furthermore, coinfected mice had significant higher levels of CRP, IL-1β, IL-6, TNF-α, IL-22 and KC as compared with PR8 or NTHi alone (Figures 7A–E, G). We also found that the levels of the adhesion molecules, including ICAM-1 and CEACAM-1, as well as the extracellular matrix Fn, were significantly higher in coinfected mice than the CS group (Figures 7I–K).

**FIGURE 8:**
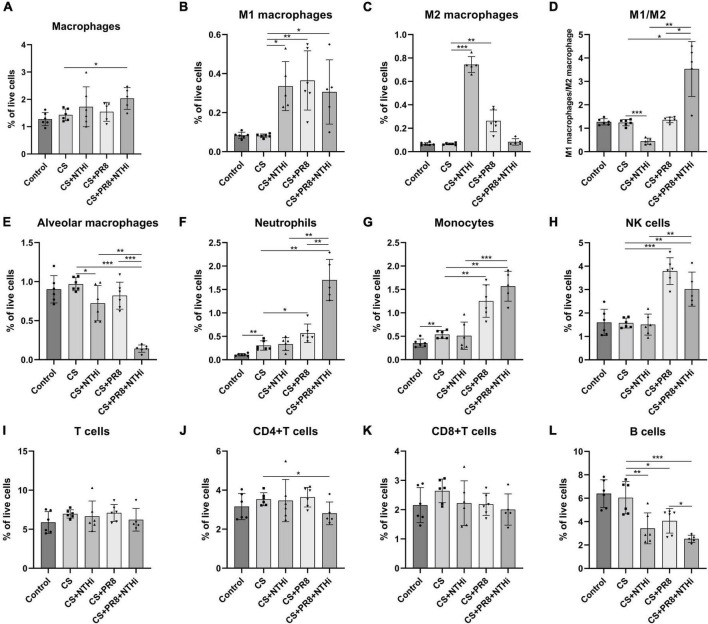
Flow cytometric analysis of innate and adaptive immune cells in AECOPD mice lungs. Following digestion of lungs, the collected cells were analyzed by flow cytometry for monocyte-derived macrophages **(A)**, M1 macrophages **(B)**, M2 macrophages **(C)**, M1 macrophages to M2 macrophages ratio **(D)**, alveolar macrophages **(E)**, neutrophils **(F)**, monocytes **(G)**, NK cells **(H)**, T cells **(I)**, CD4 + T cells **(J)**, CD8 + T cells **(K)**, and B cells **(L)**. Data are expressed as mean ± SD (*n* = 6). *p < 0.05, **p < 0.01, and ***p < 0.001.

We further performed flow cytometric analysis of inflammatory cells, including macrophages (CD45 + /CD11b + /F4/80 + /Ly6G-), M1 macrophages (CD45 + /CD11b + /F4/80 + /Ly6G-/CD86 +), M2 macrophages (CD45 + /CD11b + /F4/80 + /Ly6G-/CD206 +), alveolar macrophages (CD45 + F4/80 + CD11c + CD11b-), neutrophils (CD45 + /Ly6C + /Ly6G + /CD11b +), monocytes (CD45 + Ly6C + Ly6G-CD11b +), NK cells (NK1.1 + CD3-), T cells (CD45 + /CD3 +), CD4 + T cells (CD45 + /CD3 + /CD4 + /CD8-), CD8 + T cells (CD45 + /CD3 + /CD4-/CD8 +), and B cells (CD45 + /CD3-/CD19 +). COPD mice infected with NTHi alone had significantly increased M1 and M2 macrophages but decreased alveolar macrophages as compared with non-infected COPD mice (Figures 8B, C, E). PR8 infection alone resulted in an increase in the number of M1 and M2 macrophages, neutrophils, monocytes, and NK cells in the infected COPD mice (Figures 8B, C, F–H). In coinfected mice, total macrophages, M1 macrophages, neutrophils, monocytes, and NK cells were significantly increased (Figure 8A, B, F–H), whereas the alveolar macrophages were significantly decreased (Figure 8E). We also showed that coinfection resulted in a significant increase in the ratio of M1/M2 (Figure 8D). Regarding adaptive immune cells, coinfection significantly reduced the levels of CD4 + T cells and B cells (Figures 8J, L), but had no effect on total number of T cells or CD8 + T cells (Figures 8I, K).

### 3.6. Coinfection results in the gut microbiota dysbiosis in COPD mice

**FIGURE 9:**
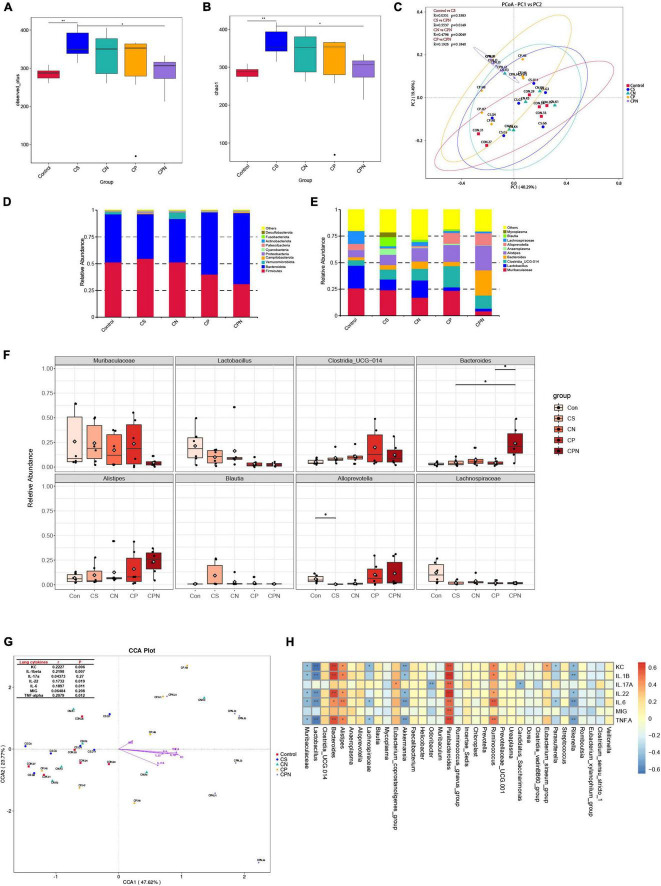
Coinfection alters gut microbiota in COPD mice. Alpha diversity indices, including observed OTUs **(A)** and Chao 1 **(B)**, for control group, CS group, CS + NTHi group (CN), CS + PR8 group (CP), and CS + PR8 + NTHi group (CPN). **(C)** PCoA of weighted UniFrac distances, with *R* and p-values calculated by Anosim. The average relative abundance of the gut microbiota at phylum **(D)** and genus **(E)** levels analyzed by Bar-plot. **(F)** Comparison of the relative abundance at the genus levels. **(G)** Correlations between cytokines and gut microbiota identified by (CCA), with mental test displayed on the lop-left corner. **(H)** Correlation between each bacteria genus and cytokine analyzed by spearman correlation tests. *p < 0.05 and **p < 0.01.

It has been reported that exposure to CS or biofuels disrupts the gut microbiota of COPD mice, which may influence disease progression (Li et al., 2021). Infection with IAV can also alter the gut microbiota, which increases susceptibility to viral infection and bacterial superinfection, exacerbates lung injury, and even increases mortality (Yildiz et al., 2018). However, the alteration of gut microbiota in AECOPD individuals remained unknown. Hence, we sequenced the 16S rRNA gene of colon contents to further investigate the effect of coinfection on the gut microbiota in COPD mice. After removing low-quantity data, 1,810,199 effective sequence reads from 30 samples were utilized for analysis and 9,403 operational taxonomic units (OTUs) were identified. The number of OTUs was significantly higher in mice exposed to CS than in the control group, but COPD mice infected with NTHi or PR8 alone had a slightly lower number of OTUs, and the decrease was even more pronounced in coinfection (Figure 9A). Chao 1 indices also showed a similar pattern of decline in alpha diversity (Figure 9B). In addition, PCoA results revealed varying degrees of shift across all groups, with the most significant shifts occurring between CS and CPN (*p* = 0.0149) and between CN and CPN (*p* = 0.0049) (Figure 9C).

We then analyzed the taxonomic level of microbial communities that may have contributed to the changes in alpha and beta diversity. At the phylum level, all colon contents contained four major bacterial phyla: *Bacteroidetes*, *Firmicutes*, *Proteobacteria*, and *Actinobacteria*. Mice exposed to CS for 14 weeks had a greater relative abundance of *Actinobacteria*, which was decreased when COPD mice were infected with NTHi (*p* < 0.05) or PR8 (*p* > 0.05) (Figure 9D). COPD mice with coinfection exhibited more pronounced decreases in *Actinobacteria* and *Firmicutes*, and an increase in *Bacteroidetes* (Figure 9D). In comparison to COPD mice, coinfected mice had lower abundance of *Muribaculaceae*, *Lactobacillus*, and *Lachnospiraceae*, and higher abundance of *Bacteroides* (Figures 9E, F).

Inflammatory cytokine production induced by acute viral respiratory infections is an important cause of gut microbiota changes (Sencio et al., 2021). We further analyzed the correlation between gut microbiota and the expression of lung cytokines. The Mantel test revealed a positive correlation between the diversity of gut microbiota and multiple inflammatory cytokines, including KC, IL-1β, IL-6, and TNF-α, which were significantly elevated in coinfected COPD mice (Figure 9G). We then investigated the association between lung cytokines and individual microbes. Interestingly. the abundance of commensal bacteria, including *Muribaculaceae*, *Lactobacillus*, *Akkermansia*, *Lachnospiraceae*, and *Rikenella*, were negatively correlated with most of cytokines. On the contrary, the higher levels of lung cytokines were correlated with increased abundance of *Bacteroides*, *Alistipes*, *Parabacteroides*, and *Ruminococcus* (Figure 9H), but the last three genus did not significantly differ among all groups.

## 4. Discussion

Respiratory viral and/or bacterial infection are the most frequently cited causes of AECOPD, and coinfection with these pathogens accounts for 25% of severe AECOPD; this percentage is likely to increase as more sensitive diagnostic tests are developed (Papi et al., 2006). Coinfection is associated with longer hospitalization and more severe lung injury in COPD patients (Papi et al., 2006). However, it is unknown precisely how viral and bacterial coinfection affects the progression of disease during an exacerbation. By exposing mice to cigarette smoke followed by viral and bacterial coinfection, it is advantageous to replicate the development and progression of AECOPD, investigate the interaction between two pathogens, and explore potential therapeutic interventions. In this study, we demonstrated that COPD mice succumbed when exposed to both influenza virus and NTHi, and that influenza virus markedly increased the host susceptibility to NTHi replication, whereas NTHi had no effect on influenza virus growth, which was consistent with our previous findings in coinfected mice without cigarette smoke exposure (Wu et al., 2021). These results suggest that a prior infection with PR8 could promote NTHi growing in the lungs of COPD mice, and the uncontrolled growth of NTHi may be one of the causes of lethality in COPD mice. It has also been reported that influenza infection increases susceptibility to and decreases elimination of other bacteria, including *S. aureus* and *S. pneumoniae* (McCullers and Rehg, 2002; Zhao et al., 2021). In addition, our AECOPD mice model not only had pathologic features of viral and bacterial pneumonia, which was consistent with our previous finding in coinfected normal mice (Wu et al., 2021), but also had emphysema, which resulted in decreased lung function.

At present, there are numerous mechanisms underlying the increased susceptibility to infections due to exposure to cigarette smoking. Firstly, cigarette exposure dampens anti-infective mechanism, such as antiviral signaling (Modestou et al., 2010; Duffney et al., 2018), pulmonary pathogen clearance (Sussan et al., 2015), and pathogen-dependent endocytosis (Duffney et al., 2020). In addition, viral infection may compromise the epithelial barrier, alter the mucosal surface environment, degrade antimicrobial peptides, and expose adhesion receptors, such as ICAM-1, CEACAM-1, platelet-activating factor receptor (PAFr), and Fn, thereby facilitating bacterial adhesion and growth (McCullers and Rehg, 2002; Bosch et al., 2013; Su et al., 2018). Therefore, higher levels of ICAM-1 and CEACAM-1 in our coinfected mice may contribute to enhanced NTHi growth.

Acute exacerbations of COPD are episodes of worsening respiratory symptoms that are always associated with increased airway and system inflammation (Wedzicha and Seemungal, 2007). Airway neutrophilia is a common feature of COPD patients and correlated with airway obstruction, decline in FEV1, and development of emphysema (Jasper et al., 2019). Although neutrophils traps pathogens and control overwhelming infections by casting neutrophil extracellular traps (NETs), massive formation of NETs may result in respiratory epithelial and endothelial cell death and accelerates disease progression, which is commonly seen in COPD (Trivedi et al., 2021). In addition, the increased migration of neutrophils in the lungs of COPD patients was frequently accompanied by decreased migration accuracy, resulting in increased “bystander” tissue damage caused by proteinase released from these cells (Sapey et al., 2011; Tregay et al., 2019). Therefore, the increased neutrophils in our CS-exposed mice and its further elevation following coinfection might contribute to the severity of AECOPD in mice. Oxidative stress-induced impairment of alveolar macrophage (AM) function reduces phagocytosis of bacteria and efferocytosis of apoptotic cells, which has been linked to increased susceptibility to AECOPD (Vlahos and Bozinovski, 2014). Although CS exposure did not affect the number of macrophages, M1 and M2 macrophages, or alveolar macrophages in our model, it is possible that the capability of macrophages to eliminate invading microbes was impaired. Our results also showed that a single NTHi or PR8 infection reduced alveolar macrophages by 25 and 15%, respectively, when compared to CS mice without infection, and that alveolar macrophages were further reduced when these two microbes were combined (Figure 8E). This was comparable to the findings of Ghoneim et al. (2013), who demonstrated that coinfection during the AM depletion phase led to significant body weight loss and mortality, and that the depletion of alveolar macrophages during influenza virus infection promoted bacterial superinfections. Macrophage can be polarized into classically activated (M1) and alternatively activated (M2) macrophages. M1 macrophages play an important role in the early stages of inflammation by phagocytosing and eliminating foreign pathogens as well as producing pro-inflammatory cytokines (Huang et al., 2018). In contrast, M2 macrophages are associated with inflammation resolution and tissue repair by reducing levels of pro-inflammatory cytokines in the cellular space (Huang et al., 2018). Balanced polarization of M1/M2 macrophage determines the lung inflammation or injury. At the stage of severe infection and inflammation, macrophages first polarized to M1 phenotype by producing TNF-α, IL-1β, IL-12, and IL-23 against the stimulus l (Shapouri-Moghaddam et al., 2018). Our findings followed a similar pattern, with coinfection causing higher M1/M2 ratio and release of TNF-α and IL-1β. Although total T cells and CD8 + T cells were not significantly different between groups, CD4 + T cells were significantly diminished in our coinfected mice compared to CS-exposed mice without infection. It has been demonstrated that CD4 + T cells play a role in the development and progression of AECOPD, and that a decrease in CD4 + T cells may be one of the contributing factors to the deterioration of COPD (Xue et al., 2022). In addition, multiple subsets of CD4 + T cells, such as regulatory T cells (Tregs), T helper 17 cells (Th17), type 1 T helper (Th1) cells, and Th2 cells, have been implicated in the pathogenesis of AECOPD (Guan et al., 2018; Wei and Sheng Li, 2018), which needs to be investigated further in our study.

Respiratory infections could result in an impaired microbiota phenotype (De Steenhuijsen Piters et al., 2016; Ferreira et al., 2020). Furthermore, it is believed that gut microbiota may increase gut permeability, allowing bacteria and toxins to enter the circulatory system and exacerbate the systemic inflammation (Mizutani et al., 2022). Our and other studies have demonstrated that altered gut microbiota composition correlated with higher levels of inflammatory cytokines (Yeoh et al., 2021; Mizutani et al., 2022). Clinical studies have demonstrated that the gut microbiota of COPD patients differs from that of healthy individuals, with fewer *Bacteroides* and more *Firmicutes* (Li et al., 2021; Chiu et al., 2022), which is similar to our finding in COPD mice. In contrast, viral infection could result in an increase in the level of *Bacteroides* and a decreased level of *Firmicutes* (Al Khatib et al., 2021), which is also similar to our finding in AECOPD mice. It has also been suggested that dysbiosis of *Bacteroidetes* and *Firmicutes* is a potential inflammatory trigger (Li et al., 2021). Changes in the *Firmicutes*/*Bacteroidetes* (F/B) ratio caused inflammatory bowel disease (IBD), and single or combined use of probiotic from the phylum *Firmicutes* were effective in restoring the gut microbial balance by influencing the F/B ratio, thereby alleviating intestinal inflammation (Stojanov et al., 2020). *Actinobacteria* have been found to colonize on the surface of respiratory mucosa of healthy individuals (Rigauts et al., 2022). We also revealed a dynamic pattern of *Actinobacteria* that increased with CS exposure but decreased after infection with PR8, NTHi, or both microbes. This could be explained by Li et al. (2022) who demonstrated a negative correlation between *Actinobacteria* and the frequency of AECOPD. *Lachnospiraceae* are the most common *Firmicutes* families that have been shown to decrease in patients with IBD and ulcerative colitis (UC) (Nagao-Kitamoto and Kamada, 2017; Vacca et al., 2020). This finding was consistent with our result in AECOPD mice. A study led by Chen found that NLRP12 could promote beneficial strains of *Lachnospiraceae*, thereby reducing intestinal inflammation (Chen et al., 2017). *Bacteroides spp*. are “friendly” commensals when they are in the gut, but when they are located elsewhere in the body, they tend to transform into opportunistic pathogens (Zafar and Saier, 2021). *Bacteroides* level increased in the gut of our coinfected mice, and this was positively correlated with cytokine expression in the lungs. It was unknown whether *Bacteroides* moved from the gut to the lung and became pathogenic. In our study, coinfected COPD mice also had decreased level of *Muribaculaceae* in the guts, which were negatively correlated with pulmonary inflammatory cytokines. This bacteria has been shown to be effective in the treatment of IAV infection (Zelaya et al., 2016). *Lactobacillus* was found to be negatively correlated with most of cytokines in our coinfected mice, and oral administration of heat-killed or plant-derived *Lactobacillus* was effective in protecting against IAV infection by enhancing T-cell factor production and IFN response (Kobayashi et al., 2011; Waki et al., 2014). According to clinical studies, *Lactobacillus* was also the most commonly used probiotic that reduced the risk of viral respiratory tract infections (Shi et al., 2021). Therefore, dysbiosis of the gut microbiota correlates with deterioration in gut and lung inflammation, and development of mono- or poly-microbial intervention may prevent the progression of gut and lung injury.

Lung microbiota is variable and distinct of those from guts (Dumas et al., 2018), and nasally infection with microbes could directly alter the lung microbiota. Bacteria colonization is observed in stable COPD patients, and respiratory viral and/or bacterial infection further leads to the development of AECOPD. It is interesting that AECOPD may be caused by new strains of bacteria rather than new families or genera; specifically, new strains of *Haemophilus influenzae* were implicated (Brennan et al., 2022). Patients with stable COPD were reported to have *Firmicutes* (31.63%), *Bacteroidetes* (28.94%), and *Proteobacteria* (19.68%) in lungs, whereas those with AECOPD had a dominant bacterial phylum of *Proteobacteria* (30.29%), *Firmicutes* (29.85%), and *Bacteroidetes* (14.02%) (Russo et al., 2022). In a Chinese cohort study, *Streptococcus* was found to be the most predominant genus in sputum of AECOPD patients (Wang et al., 2020). *Pasteurellaceae*, *Fusobacterium*, *Solobacterium*, *Haemophilus*, *Atopobium*, *Corynebacterium*, and *Streptococcus* were also found to be enriched in the sputum microbiomes of eosinophilic AECOPD (Wang et al., 2020). By sampling endotracheal aspirates from AECOPD individuals, *Firmicutes* and *Proteobacteria* was also found to be the most abundant bacterial phyla, and *Streptococcus* was the most abundant bacterial species (Sun et al., 2020). In an emphysema mouse model, elastase induces a decline in microbiota richness and diversity, with *Pseudomonas* and *Lactobacillus* genera increasing and *Prevotella* decreasing (Wang et al., 2017). IAV causes subtle, transient changes in the microbial composition of the lower respiratory tract of mice, with *Lactobacillus* dominating (Yildiz et al., 2018). Mice infected with *S. pneumoniae* after 6 months of exposure to cigarette smoke had decreased pulmonary microbiota diversity and increased *Lactobacillaceae* levels in the upper respiratory tract (Hilty et al., 2020). However, it remains unclear how changes in the composition of lung microbiome in COPD mice with NTHi infection or IAV-NTHi coinfection. Furthermore, the underlying mechanism driving these changes in lung microbiota observed in patients and animals should be investigated further in our coinfection model.

## 5. Conclusion

Coinfection with H1N1 and NTHi increases disease severity in COPD mice due to increased lung inflammation, which is correlated with a dysbiosis of gut microbiota.

## Data availability statement

The datasets presented in this study can be found in online repositories. The names of the repository/repositories and accession number(s) can be found in this article/supplementary material.

## Ethics statement

The animal study was reviewed and approved by the Animal Care and Use Committee of Kunming Medical University.

## Author contributions

Z-FY, W-MY, and X-SX were the principal investigators who conceived the scientific idea. XW, BL, CX, K-LM, H-XZ, D-YZ, SC, Y-CW, R-FC, H-MJ, L-PC, L-ZD, and P-FX carried out the experiments. JZ was responsible for pathological examine. XW and R-FL contributed to the experimental design, data analysis, and article writing. Z-SL and R-FL reviewed and revised the manuscript. All authors have read and agreed to the submitted version of the manuscript.
